# Beyond Static Assessment: A Proof-of-Concept Evaluation of Functional Data Analysis for Assessing Physiological Responses to High-Intensity Effort

**DOI:** 10.3390/jfmk11020151

**Published:** 2026-04-10

**Authors:** Adrian Odriozola, Cristina Tirnauca, Adriana González, Francesc Corbi, Jesús Álvarez-Herms

**Affiliations:** 1 Hologenomiks Research Group, Department of Genetics, Physical Anthropology and Animal Physiology, University of the Basque Country (UPV/EHU), 48940 Leioa, Spain; adriana.gonzalez@ehu.eus; 2 Institut Nacional d’Educació Física de Catalunya (INEFC), Centre de Lleida, Universitat de Lleida (UdL), 25192 Lleida, Spain; jesusah80@gmail.com; 3 Departamento de Matemáticas, Estadística y Computación, Universidad de Cantabria, 39005 Santander, Spain; cristina.tirnauca@unican.es; 4 Department of Clinical Sciences, Faculty of Medicine and Health Sciences, University of Barcelona, 08907 L’Hospitalet de Llobregat, Spain; f@corbi.neoma.org; 5 Phymo^®^ Lab, Physiology, and Molecular Laboratory, 40170 Collado Hermoso, Spain

**Keywords:** functional data analysis, physiological recovery, precision medicine, cycling performance, unsupervised clustering, responder phenotyping, lactate kinetics

## Abstract

**Background**: Conventional analyses of physiological recovery often rely on discrete metrics that assume independence across time points, thereby ignoring intrinsic temporal continuity and masking substantial interindividual heterogeneity. This proof-of-concept study assesses the efficacy of Functional Data Analysis (FDA) as a promising framework for characterizing individual response dynamics following a functional threshold power (FTP) test. **Methods**: Physiological time-series data (including blood lactate, heart rate, blood pressure, and glucose levels) collected from 21 trained cyclists (10 professionals, 11 amateurs) were represented as functional objects using FDataGrid on the original sampling grid (0, 3, 5, 10, 20 min), without basis expansion or smoothing. We conducted unsupervised functional clustering (K-means; Fuzzy K-means) and supervised classification (Maximum Depth with Modified Band Depth, K-Nearest Neighbors, Nearest Centroid, functional QDA with parametric Gaussian covariance). Model performance was estimated via Repeated Stratified 5-Fold Cross-Validation with 10 repetitions (50 folds), reporting accuracy, balanced accuracy (mean ± SD), 95% CIs, permutation *p*-values, and sensitivity/specificity from aggregated confusion matrices. **Results**: Lactate (CL) and diastolic blood pressure (DBP) provided useful and statistically significant discrimination across several classifiers (e.g., KNN, Nearest Centroid, functional QDA), whereas heart rate showed modest discriminative value and glucose intermediate performance. Unsupervised analyses revealed distinct lactate recovery profiles and graded membership for hemodynamic/metabolic variables, supporting the value of FDA for resolving heterogeneity beyond group-average trends. **Conclusions**: FDA offers a feasible and informative approach for classifying recovery phenotypes while preserving temporal structure. Findings are promising but should be interpreted with caution due to the small sample size, sparse time points, and the need for external validation in larger, independent cohorts before translation into routine decision-making.

## 1. Introduction

### 1.1. Interindividual Variability in Physiological Responses to Endurance Exercise

It is widely recognized that a standardized endurance exercise stimulus does not elicit uniform physiological adaptations across all individuals. While group-level averages consistently demonstrate the efficacy of endurance training for improving cardiorespiratory and metabolic health, these aggregate trends mask a highly heterogeneous reality at the individual level [1]. Research from the HERITAGE Family Study initially established that identical exercise prescriptions could lead to significant adaptations in some individuals classified as high responders, while eliciting minimal or even adverse changes in others regarded as low or non-responders. This phenomenon has been observed across various phenotypes, including maximal oxygen uptake, insulin sensitivity, and blood pressure [1,2].

Recent evidence has refined this paradigm, shifting focus from responder classification toward understanding the biological and methodological origins of this variability. Several systematic reviews and large-scale trials indicate that interindividual heterogeneity is not merely random noise but reflects a complex interaction between baseline physiological capacity and temporal regulatory dynamics [3,4]. Recent methodological critiques suggest that conventional analyses often overestimate the prevalence of non-responders by failing to distinguish physiological heterogeneity from measurement error and within-subject biological variation [5,6]. This distinction is particularly relevant for acute and short-term recovery dynamics in which the temporal structure of the response encodes key information about individual homeostatic regulation.

In this context, variability is influenced not only by the magnitude of change in peak values but also by the kinetics of the response, which, in practical terms, reflects the speed and efficiency of individual recovery. Therefore, capturing interindividual differences requires analytical methods that examine structured physiological dynamics rather than relying on static pre- and post-comparison approaches that overlook the temporal complexity inherent in physiological responses [7].

### 1.2. Limitations of Group-Based Analyses and Discrete Summaries

Standard group-based methodologies, such as repeated-measures ANOVA or Student’s *t*-tests, emphasize central tendencies and implicitly regard individual deviations from the group mean as error rather than a biological signal. While statistically convenient, these methodologies are fundamentally inadequate for capturing the temporal architecture of physiological responses. By converting continuous dynamic processes into discrete scalar summaries, such as average and peak values or time-to-exhaustion, traditional analyses discard critical information regarding the trajectory’s shape, rate of change, and complexity, features that frequently serve as the most sensitive indicators of physiological regulation [7,8].

Furthermore, conventional multivariate techniques such as multiple regression assume that observations are independent, a condition that is systematically violated in time-series data, where successive measurements are intrinsically autocorrelated [9].

Hence, applying static linear models to such dynamic, autocorrelated data may yield biased parameter estimates and increased type I error rates attributable to temporal noise rather than genuine physiological coupling [5,7]. Accordingly, the limitation of current research resides not solely in the paucity of data, but also in the discrepancy between the dynamic nature of physiological adaptation and the static nature of the statistical tools employed for analysis. This discordance underscores the need to adopt frameworks designed to model functions rather than discrete points.

### 1.3. Functional Data Analysis as an Alternative Framework

Functional Data Analysis (FDA) represents a paradigm shift in the statistical examination of temporal processes. Unlike multivariate statistics, which operate on finite vectors in which variable reordering is frequently permissible, FDA treats the data as continuous curves in an infinite-dimensional space, where the inherent temporal order is fundamental [10].

This conceptual transition addresses the limitations of standard group-based approaches by explicitly modeling the intrinsic continuity and autocorrelation of physiological systems. As highlighted in recent biomechanics reviews, the FDA enables researchers to analyze movement comprehensively and to view recovery as an ongoing process, rather than segmenting these phenomena into isolated snapshots [11].

Methodologically, the FDA workflow typically begins with the continuous representation of discrete data, a process that can be approached differently depending on the nature of the measurements. For datasets characterized by high-frequency recordings or substantial noise, smoothing techniques and basis expansions (e.g., B-splines or Fourier bases) are typically employed to isolate the underlying biological signal [12]. Conversely, when physiological metrics are captured at precise, standardized time intervals with high reliability, functional trajectories can be constructed directly upon the original measurement grid. It can help preserve the exactitude of the raw values, ensuring that subsequent functional analyses reflect the strictly observed data without introducing artificial variance or bias arising from functional approximation.

Once the data are functionalized, advanced exploratory techniques, such as Functional Principal Component Analysis or unsupervised functional clustering, can be applied. These methodologies allow researchers to decompose the variability in the curves into orthogonal modes or distinct groups, thereby revealing the predominant temporal patterns that characterize the population and, for example, distinguishing between delayed and rapid recovery profiles. This methodology has recently been termed Data Analysis 2.0 in fields such as continuous glucose monitoring, where the emphasis has shifted from mean values to the examination of fluctuation patterns and rates of change [13]. Adopting this perspective enables quantification of interindividual heterogeneity in the shape, timing, and magnitude of physiological responses.

### 1.4. Aim of the Study

Based on the emerging Data Analysis 2.0 framework, the central research question of this proof-of-concept study is whether the FDA can overcome the limitations of traditional discrete frameworks to capture physiological heterogeneity better. To address this, the research advances three aligned methodological objectives: (1) to quantify the information loss associated with reducing continuous physiological time-series to static metrics; (2) to validate the capacity of unsupervised functional clustering to identify distinct recovery phenotypes; and (3) to provide a reproducible analytical workflow for continuous data in sports science.

## 2. Materials and Methods

### 2.1. Study Design and Dataset

We conducted a secondary methodological analysis utilizing a verified physiological dataset originally collected from 22 trained cyclists. However, one participant was excluded from the present analysis because this individual lacked complete measurements for all the variables analyzed in this article, resulting in a final sample of 21 cyclists (10 professionals, 11 amateurs). The complete experimental design, participant characteristics, and inclusion criteria are detailed in the primary publication [14]. In brief, participants performed a maximal 20-min functional threshold power (FTP) test under controlled environmental conditions. The dataset comprises physiological time-series, specifically heart rate (HR), systolic (SBP) and diastolic blood pressure (DBP), blood lactate, and blood glucose, recorded at standardized time points (0, 3, 5, 10, and 20 min) during the recovery phase. Crucially, for the present analysis, the raw anonymised data were reused without modification to the experimental values. The study adhered to the ethical principles outlined in the Declaration of Helsinki [15] and the Spanish Biomedical Research Law 14/2007, the European Regulation (EU) 2016/679 on data protection (GDPR). Furthermore, the study aligns with methodological principles that encourage benchmarking new analytical tools against verified experimental datasets [11]. The protocol was approved by the Human Research Ethics Committee of the University of the Basque Country (M10 2021 191).

### 2.2. Conventional Discrete Variable Analysis

To establish a methodological benchmark reflecting standard practices in exercise physiology [16], recovery dynamics were analyzed using conventional polynomial regression. To characterize temporal trends at the group level, recovery dynamics were modeled using linear (y = β_0_ + β_1_t), quadratic (y = β_0_ + β_1_t + β_2_t^2^), and cubic (y = β_0_ + β_1_t + β_2_t^2^ + β_3_t^3^) regression terms, fitted to the fixed experimental time points (0, 3, 5, 10, and 20 min). Differential recovery kinetics between professional and amateur groups were assessed by comparing the regression slopes (β) and goodness-of-fit (R^2^) of the respective models. All conventional analyses were conducted utilizing GraphPad Prism (version 10.0.0, GraphPad Software, Boston, MA, USA). Statistical significance was established at *p* < 0.05. This section delineates the static control condition, representing the standard analytical resolution currently available in the field, against which the functional framework is assessed.

### 2.3. Functional Data Analysis

Functional Data Analysis was employed to address the limitations of discrete metrics and to evaluate the distribution of continuous recovery profiles [10]. In brief, this workflow translates discrete data points into continuous curves, groups these curves by overall shape (unsupervised phase), and tests whether these curve shapes can predict the athlete’s competitive level (supervised phase). The computational workflow was implemented in Python 3.13.12 using the scikit-fda package version 0.10.1 [17] and comprised two distinct phases. All physiological time series were represented as functional objects using the FDataGrid structure from scikit-fda, preserving the original sampling grid (0, 3, 5, 10, 20 min). No smoothing, basis expansion, or penalized regularization was applied at any stage; analyses operated directly on the raw discrete observations. Time was kept in physical minutes (not rescaled), and no normalization or standardization was applied to the functional objects beyond the internal computations of each classifier. This absence of time rescaling preserves exact temporal distances, which is an explicit structural requirement for the Gaussian parametric covariance used in the QDA classifier. Consequently, both the unsupervised analyses and the supervised classification models operated on the same raw functional grids. This setup guarantees that the workflow is fully reproducible, ensuring that all results reflect the empirical recovery trajectories rather than properties introduced by preprocessing or functional approximation.

In the unsupervised phase, an exploratory clustering analysis was performed to identify latent recovery patterns without prior labeling, and solutions were tested for 2, 3, and 4 clusters (κ = 2, 3, 4). Initially, the K-means algorithm was applied, which determines centroids based on geometric proximity [18]. Complementarily, the Fuzzy K-means algorithm was applied to quantify each individual’s degree of membership, facilitating the identification of overlapping physiological profiles [19].

In the second analytical phase, supervised classification was employed to identify the most informative continuous predictors of cyclist status (Professional versus Amateur). Four classification techniques were assessed: Maximum Depth Classifier, Nearest Centroid Classifier, K-Nearest Neighbors (KNN) [20], and Parameterized Functional Quadratic Discriminant Analysis (QDA). Notably, the Maximum Depth Classifier was used to assess the centrality of curves within the distribution, providing a robust metric distinct from distance-based centroids. To ensure robust model validation and minimize the influence of sampling variability inherent to small datasets, we employed a Repeated Stratified 5-Fold Cross-Validation procedure. In this framework, the data were partitioned into five stratified folds, preserving the proportion of professional and amateur cyclists. Each model was trained on four folds and evaluated on the remaining fold, cycling through all possible train–test combinations. This entire 5-fold process was then repeated 10 times with different fold assignments using a fixed random seed (0) to guarantee reproducibility. The resulting 50 validation scores (10 repeats × 5 folds) were used to compute the mean and standard deviation of accuracy and balanced accuracy, along with the associated 95% confidence intervals and permutation-based significance tests.

Before presenting the functional clustering and classification results, we first provide an illustrative group-average baseline using conventional polynomial regression. This baseline is included solely to contextualize the overall behavior of the variables at the population level and to contrast it with the individual-level structure revealed by FDA.

## 3. Results

### 3.1. Illustrative Group-Average Baseline Assessment

As an illustrative group-average baseline, we fitted polynomial models to the mean trajectories at 0, 3, 5, 10, and 20 min to summarize overall recovery trends. Because group-average regression inherently smooths over interindividual variability, this baseline serves only as a reference for visualizing overall trends. It is not intended as a competing conventional alternative to the FDA workflow.

The initial analysis using conventional polynomial regression yielded statistically significant models for all physiological variables (*p* < 0.001). As summarized in Table 1, the transition from linear to quadratic and cubic models systematically enhanced the goodness-of-fit. Notably, cubic polynomial models yielded the highest coefficients of determination (R^2^) across both groups, with values ranging from 0.60 to 0.89.

However, a more detailed analysis uncovers a significant limitation. While variables such as Heart Rate (R^2^ = 0.89) and Systolic Blood Pressure (R^2^ = 0.83) demonstrate a strong statistical fit to the group average, other metrics showed moderate to low fits, including Glucose (R^2^ = 0.79) and Diastolic Blood Pressure (R^2^ = 0.85). Critically, metabolic markers, such as Lactate levels in professionals, exhibit a markedly poorer fit (R^2^ = 0.60), even when employing the most sophisticated cubic model. This substantial unexplained variance indicates that traditional regression methods, although effective in describing general trends, are inadequate in capturing the biological complexity and individual heterogeneity inherent in the recovery process, often interpreting distinct responder patterns as mere statistical noise.

While this group-based baseline provides a convenient descriptive overview, it cannot resolve the substantial interindividual variation present in the recovery dynamics. Therefore, we next applied Functional Data Analysis, beginning with unsupervised clustering, to examine the underlying structure of individual trajectories and identify physiologically meaningful recovery phenotypes.

### 3.2. Unsupervised Profiling: Identification of Physiological Phenotypes

**Figure 1:**
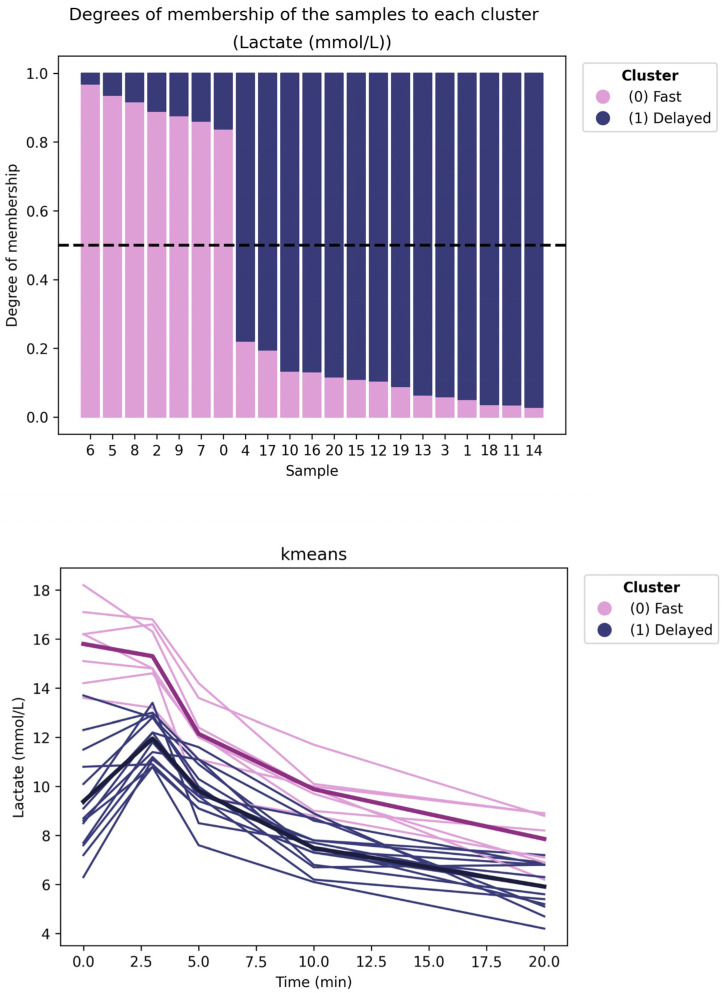
Unsupervised functional clustering of lactate recovery dynamics. The upper panel presents the Fuzzy K-Means analysis, showing the membership probability (ranging from 0 to 1) of each individual to a particular cluster. The lower panel displays the resulting K-Means classification curves, where bold lines represent the mean functional trajectory (centroid) of each cluster, which identifies two distinct physiological phenotypes: “Fast Recovery” (Cluster 0) and “Delayed Recovery” (Cluster 1). Sample IDs 0–9 correspond to professional cyclists, while IDs 10–20 correspond to amateurs.

Overall, Functional Data Analysis uncovered distinct recovery profiles that traditional regression methods failed to detect. Specifically, applying unsupervised clustering algorithms (K-means and Fuzzy K-means) to the continuous lactate curves enabled the identification of two clearly differentiated responder dynamics, as shown in Figure 1.

The initial cluster (Cluster 0) corresponds to a fast-recovery phenotype, characterized by a rapid, exponential decline in blood lactate immediately after exertion. Conversely, the subsequent cluster (Cluster 1) exhibits a delayed recovery pattern, retaining elevated lactate levels for an extended period before stabilization. Notably, while the fast Recovery group was exclusively composed of professionals, the delayed phenotype comprised the entire amateur cohort, along with a specific subset of professionals (with IDs 1, 3, and 4) exhibiting atypical, slower clearance rates. Significantly, the Fuzzy K-means algorithm corroborated that this delineation is robust, with participants exhibiting high membership degrees (>0.80) in their respective clusters, indicating a clear physiological distinction.

**Figure 2:**
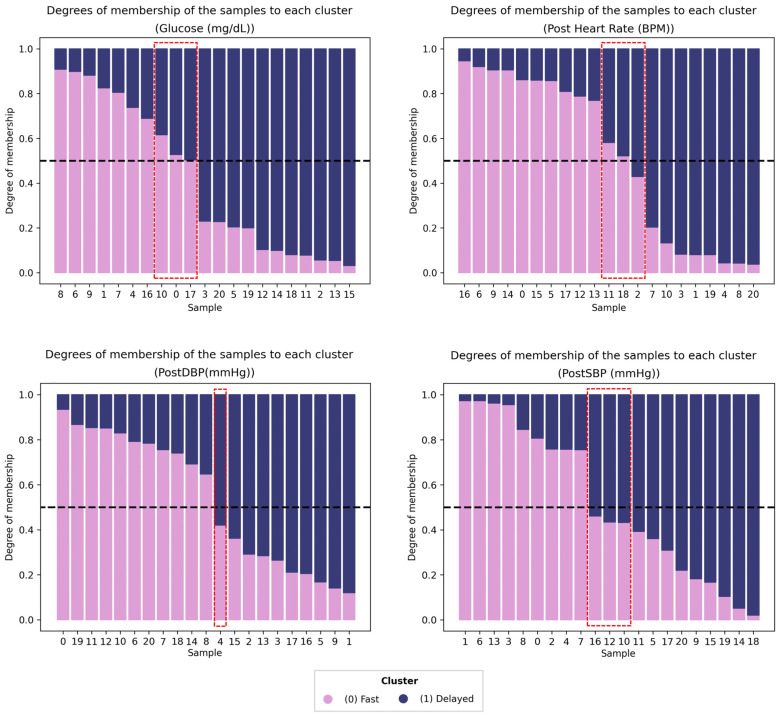
Analysis of the biological continuum in hemodynamic and metabolic recovery using Fuzzy K-Means. The plots display the degree of membership for glucose, heart rate, diastolic blood pressure (DBP), and systolic blood pressure (SBP). In contrast to the distinct separation observed in lactate dynamics, these variables exhibit significant phenotypic overlap. Sample IDs 0–9 correspond to professional cyclists, while IDs 10–20 correspond to amateurs. Individuals exhibiting intermediate membership in a single physiological profile (mixed phenotype) are highlighted in red dashed boxes, indicating hybrid recovery dynamics that resist binary classification.

Nevertheless, this pronounced dichotomy was not uniformly observed across all variables. As illustrated in Figure 2, variables such as Heart Rate, Glucose, and Blood Pressure (SBP and DBP) demonstrated more intricate, overlapping structures. In these instances, the Fuzzy algorithm proved particularly elucidative, revealing hybrid physiological behaviors in which individuals denoted by red dashed boxes exhibited partial membership across multiple profiles. This indicates that, although lactate dynamics tend to follow a relatively binary pattern within this cohort, hemodynamic and metabolic recovery often lie on a continuum that defies strict classification into discrete categories.

### 3.3. Supervised Classification: Predictive Accuracy of Functional Dynamics

Beyond unsupervised profiling, supervised learning techniques were employed to identify the most reliable physiological trajectories for predicting cyclist status (Professional vs. Amateur). The predictive efficacy of four functional classification algorithms is summarized in Table 2.

The cross-validated results revealed substantial variability in the discriminative capacity of the different recovery trajectories (Table 2). Across classifiers, Lactate (CL) and Diastolic Blood Pressure (DBP) consistently showed the strongest predictive value, yielding the highest mean accuracy and balanced accuracy estimates under Repeated Stratified 5-Fold Cross-Validation (10 repetitions, 50 folds). Models such as K-Nearest Neighbors, Nearest Centroid, and functional QDA achieved high, statistically significant performance on these variables, with narrow confidence intervals and permutation *p*-values generally below 0.01, indicating performance above chance.

In contrast, heart rate (HR), despite its high goodness-of-fit in the group-average regression analysis, showed modest classification performance, with wide variability and mostly non-significant permutation tests across models. This discrepancy reflects the fact that HR recovery follows a broadly similar decay pattern across cyclists, providing limited discriminatory texture compared with metabolic (Lactate) or hemodynamic (DBP) dynamics. Glucose (GLUC) showed intermediate performance, with classification accuracy and balanced accuracy estimates consistently lower than those obtained for lactate or DBP.

## 4. Discussion

Our baseline analysis demonstrated that conventional polynomial regression achieves an excellent statistical fit (R^2^ > 0.80) when applied to group-averaged data. Superficially, this suggests that variables such as Heart Rate and Systolic Blood Pressure follow predictable recovery trajectories. However, these results reveal a methodological paradox: the model accurately captures the aggregate mean and characterizes the general physiological response, yet it cannot account for the biological heterogeneity observed at the individual level. This aligns with prior work on inter-individual variability, in which aggregate analyses may mask distinct responder phenotypes, thereby limiting their value for individualized monitoring.

This phenomenon, known as the fallacy of the average [21], aligns with established research on interindividual variability, indicating that aggregate analyses frequently mask diverse responder phenotypes [1,2,4], thereby limiting the utility of conventional models for precision monitoring.

Methodologically, this oversimplification arises because conventional regression smooths out fluctuations and treats residuals as error, imposing a statistical homogeneity that does not exist biologically. As observed in our dataset, while cubic models captured the general monotonic decay, they remained blind to subtle temporal deviations, such as delayed stabilization or secondary peaks, that characterize the actual physiological stress response. Consequently, relying solely on discrete metrics or averaged curves risks categorizing athletes based on a statistical artifact rather than their true biological status, potentially leading to misclassified training prescriptions or overlooked maladaptations.

In parallel, traditional discrete analyses often force athletes into rigid categories, such as high vs. low responders or positive vs. negative adaptations, based on arbitrary cut-off points [1,22]. Our results challenge this binary approach, demonstrating the promising ability of functional clustering to resolve the intermediate phenotypes of physiological recovery. By capturing the full continuous spectrum of the data, the FDA overcomes the limitations of biological reductionism, aligning with recent reviews advocating for continuous profiling over categorical labeling [5].

Furthermore, our functional analysis exposed a pronounced divergence in the architectural complexity of recovery. Specifically, lactate dynamics exhibited a sharp dichotomous structure, facilitating clear phenotypic separation (Figure 1). In contrast, hemodynamic variables such as Heart Rate and Blood Pressure followed a continuous, overlapping distribution (Figure 2). This contrast likely reflects the physiological distinction between specific local metabolic clearance pathways and the systemic nature of hemodynamic regulation, which acts as a global integrator of autonomic, hormonal, and thermoregulatory inputs [23,24]. This physiological dichotomy directly underpins the classification performance observed in our supervised analysis. Despite the high statistical fit of the regression models, Heart Rate lacked discriminatory power in predicting cyclist status. This apparent contradiction aligns with the understanding that cardiac recovery is governed by generic autonomic reactivation patterns, which, while faster in professionals, follow a similar exponential decay across all trained cyclists [25]. Consequently, while HR remains valid for monitoring general internal load, it lacks the specific shape texture necessary to distinguish complex biological phenotypes.

Under Repeated Stratified 5-Fold Cross-Validation with 10 repetitions (50 folds), we observed considerable variability in discriminative capacity across variables and classifiers. Lactate and diastolic blood pressure yielded high, statistically significant cross-validated performance with several methods (e.g., K-Nearest Neighbors, Nearest Centroid, functional QDA), accompanied by relatively narrow confidence intervals and significant permutation tests—indicating performance above chance in this dataset. In contrast, Heart Rate showed modest classification performance despite strong goodness-of-fit at the group-average level, consistent with its broadly similar decay pattern across trained cyclists; glucose showed intermediate performance. Together, these findings suggest that curve-shape information from selected variables can serve as feasible markers for classifying athlete status, complementing traditional descriptive analyses.

Unsupervised analyses further reinforced this pattern. Lactate trajectories displayed two clearly distinguishable recovery phenotypes. At the same time, hemodynamic and glucose responses exhibited graded membership that was better captured by Fuzzy K-means, a well-suited option when profiles may overlap. This observation fits the physiological logic that local metabolic clearance and vascular resistance dynamics can encode sharper kinetic differences than global hemodynamic integrators, which tend to show smoother, shared decay forms.

Methodologically, our workflow prioritized parsimony and reproducibility. We represented time series as functional curves on the original grid without applying smoothing or basis expansion, and we validated models using repeated, stratified cross-validation to reduce the influence of idiosyncrasies arising from a single split. Reporting accuracy, balanced accuracy (mean ± SD), 95% CIs, permutation *p*-values, and sensitivity/specificity (from fold-aggregated confusion matrices) provides a more reliable summary for small samples. It clarifies which effects are statistically meaningful rather than contingent. These findings should be interpreted with caution, given the small sample size, the limited number of time points, and the need for external validation in larger, independent cohorts before broader application. While some specific train/test partitions may yield perfect scores, the repeated cross-validation estimates provide the appropriate summary for this proof-of-concept dataset.

While our previous research using discrete time points successfully identified general responder profiles [14], the functional approach employed here reveals that the diagnostic value lies not merely in the magnitude of the drop, but in the shape derivatives of the curve. By treating these variables as continuous functions, the model detects subtle kinetic variations, likely reflecting the efficiency of monocarboxylate transporters and mitochondrial density [26], as well as the dynamics of vascular resistance [27,28], which remain invisible to static, point-based metrics.

To address the structural heterogeneity observed in the hemodynamic variables, the application of Fuzzy C-Means emerged as a critical methodological framework for quantifying biological ambiguity. By assigning a continuous degree of membership, the algorithm successfully identified athletes situated in the transitional zones of the physiological spectrum as intermediate phenotypes.

From a translational perspective, these individuals represent a pivotal challenge for precision monitoring, as a conventional binary approach would inevitably misclassify them as outliers or force them into an ill-fitting category. Recognizing this transitional status is fundamental to advancing Precision Medicine in sports, shifting the focus from rigid group-based templates to interventions tailored to the athlete’s evolving position along the biological continuum [4,29].

### Practical Applications: From Data to Decision

**Figure 3:**
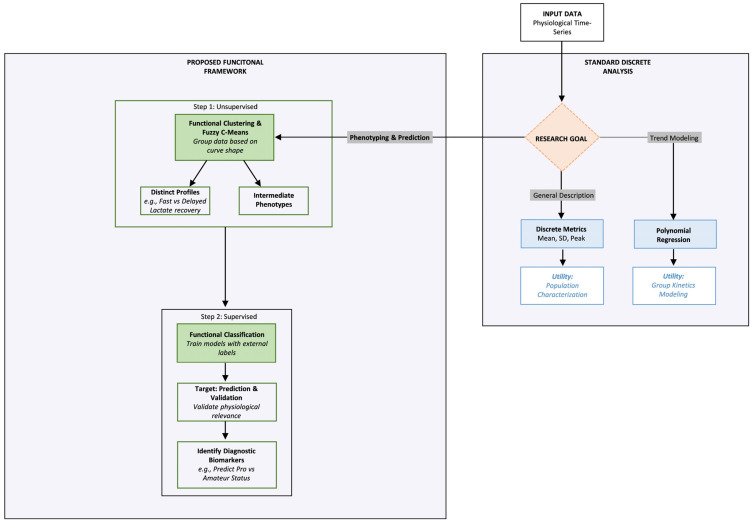
The Methodological Road Map: A decision tree for physiological recovery analysis. This diagram illustrates the optimal analytical workflow based on the research objective. (Right Panel) Standard discrete analysis: remains the method of choice for general description and trend modeling in homogeneous populations, though it relies on fixed mathematical laws that may mask individual variability. (Left Panel) Proposed functional framework: essential for complex phenotyping, following a sequential logic: Step 1 (Unsupervised discovery): uses functional clustering to reveal the full data structure, identifying both distinct profiles (e.g., Fast vs. Delayed Lactate recovery) and, via Fuzzy K-Means, intermediate phenotypes in transitional zones. Step 2 (Supervised application): converges these findings to validate physiological relevance and identify predictive biomarkers that distinguish athlete status with high diagnostic accuracy.

Based on the evidence presented, we argue that the primary limitation of current sports science research lies in the analytical workflow rather than the data collection. To address the constraints of the prevailing regression-centric paradigm, which frequently conflates biological variability with statistical noise [1,21]. We propose a reordered analytical framework conceptualized as a Methodological Road Map (Figure 3) that prioritizes pattern recognition over simple aggregation.

As illustrated in Figure 3, researchers should view the analytical process as a decision tree contingent on the specific research goal. When the objective is limited to general description or trend modeling in homogeneous populations, traditional discrete metrics such as mean, standard deviation, and peak, as well as polynomial regression, remain valid and efficient tools [16]. This approach is particularly suitable for testing known mathematical laws, such as exponential decay [6]. However, its fundamental limitation lies in its static resolution, which reduces complex recovery curves to scalar metrics and, therefore, inevitably masks the temporal structure of recovery and dilutes individual variability [7].

In contrast, for phenotyping and Precision Medicine, where resolving biological heterogeneity is fundamental, the functional framework (left panel, Figure 3) offers a promising alternative by treating recovery as a continuous curve in an infinite-dimensional space [10]. This framework operates through a critical, sequential logic, with Unsupervised FDA as the first line of analysis. Before applying predictive models, this discovery phase is essential to reveal the full data structure, identify atypical temporal patterns, and uncover latent subgroups that standard metrics miss [5,11]. Furthermore, to address the biological ambiguity observed in systemic variables such as HR, the application of Fuzzy K-Means is a well-suited standard. Unlike rigid clustering, this algorithm quantifies membership, enabling precise identification of intermediate profiles in the transitional zones of adaptation [2,13].

Once the underlying structure is defined, comprising both distinct and intermediate phenotypes, the workflow advances to supervised application. In this validation phase, the precise functional shape of the response serves as a diagnostic biomarker to identify the physiological determinants of elite performance. Specifically, the model’s ability to distinguish between athlete statuses with high accuracy validates that these temporal dynamics are not merely descriptive but are critical discriminators of physiological capacity [12]. Ultimately, adopting this functional workflow provides a more robust analytical lens, ensuring that the intrinsic temporal dynamics of recovery are preserved and utilized for precision decision-making.

## 5. Conclusions

This proof-of-concept indicates that Functional Data Analysis is a promising and feasible framework for classifying recovery phenotypes by preserving the temporal structure of physiological responses. Under repeated stratified 5-fold cross-validation, lactate and diastolic blood pressure provided useful discriminative information, whereas heart rate showed modest performance and glucose intermediate results. Taken together, these findings support the value of FDA as a complementary approach to group-average descriptive analyses when the goal is to resolve individual heterogeneity. These findings should be interpreted with caution, given the small sample size, the limited number of time points, and the need for external validation in larger, independent cohorts before broader application.

## Figures and Tables

**Table 1:** Goodness-of-fit comparison (R^2^) across Linear, Quadratic, and Cubic regression models for physiological recovery dynamics.

Variable	Group	Linear R^2^	Quadratic R^2^	Cubic R^2^	Sig. (*p*)
Lactate	Amateur	0.56	0.56	**0.72**	<0.001
	Professional	0.55	0.57	**0.60**	<0.001
Heart Rate	Amateur	0.81	**0.89**	0.89	<0.001
	Professional	0.70	**0.83**	0.83	<0.001
Glucose	Amateur	0.64	**0.70**	0.70	<0.001
	Professional	0.63	**0.79**	0.79	<0.001
Systolic BP	Amateur	0.46	0.72	**0.76**	<0.001
	Professional	0.53	0.82	**0.83**	<0.001
Diastolic BP	Amateur	0.44	**0.69**	0.69	<0.001
	Professional	0.65	**0.85**	0.85	<0.001

Data show the coefficient of determination (R^2^) for Linear (1st-order), Quadratic (2nd-order), and Cubic (3rd-order) regression models fitted to the group-averaged time-series data. Bold values indicate the model with the highest R^2^ achieved for each variable within the respective group. All models were statistically significant (*p* < 0.001). Abbreviation: BP, Blood Pressure.

**Table 2:** Cross-validated performance by classifier and variable.

Classifier	Metric	CL	GLUC	PostDBP	PostSBP	PostHR
Maximum Depth	Accuracy (mean ± SD)	0.813 ± 0.174	0.656 ± 0.206	0.577 ± 0.263	0.728 ± 0.233	0.437 ± 0.178
	Balanced Acc (mean ± SD)	0.810 ± 0.180	0.660 ± 0.203	0.582 ± 0.264	0.730 ± 0.230	0.442 ± 0.179
	95% CI (Accuracy)	[0.764, 0.862]	[0.597, 0.715]	[0.502, 0.652]	[0.662, 0.794]	[0.387, 0.487]
	*p*-value (Acc/Bal)	0.001/0.001	0.062/0.056	0.209/0.203	0.018/0.018	0.730/0.712
	Sens/Spec	0.800/0.818	0.690/0.618	0.590/0.573	0.720/0.736	0.620/0.273
KNN	Accuracy (mean ± SD)	0.860 ± 0.165	0.782 ± 0.192	0.872 ± 0.160	0.802 ± 0.164	0.515 ± 0.248
	Balanced Acc (mean ± SD)	0.850 ± 0.175	0.780 ± 0.194	0.872 ± 0.161	0.800 ± 0.165	0.513 ± 0.251
	95% CI (Accuracy)	[0.813, 0.907]	[0.728, 0.836]	[0.826, 0.918]	[0.755, 0.849]	[0.445, 0.585]
	*p*-value (Acc/Bal)	0.001/0.001	0.007/0.007	0.001/0.001	0.002/0.003	0.404/0.416
	Sens/Spec	0.700/1.000	0.730/0.827	0.810/0.936	0.690/0.909	0.410/0.618
Nearest Centroid	Accuracy (mean ± SD)	0.860 ± 0.165	0.756 ± 0.210	0.830 ± 0.224	0.838 ± 0.165	0.568 ± 0.246
	Balanced Acc (mean ± SD)	0.850 ± 0.175	0.755 ± 0.210	0.832 ± 0.223	0.838 ± 0.164	0.570 ± 0.248
	95% CI (Accuracy)	[0.813, 0.907]	[0.696, 0.816]	[0.766, 0.894]	[0.791, 0.885]	[0.498, 0.638]
	*p*-value (Acc/Bal)	0.001/0.001	0.013/0.014	0.002/0.002	0.001/0.001	0.291/0.284
	Sens/Spec	0.700/1.000	0.710/0.800	0.820/0.845	0.800/0.873	0.580/0.564
Functional QDA	Accuracy (mean ± SD)	0.788 ± 0.180	0.794 ± 0.194	0.872 ± 0.144	0.857 ± 0.176	0.647 ± 0.247
	Balanced Acc (mean ± SD)	0.788 ± 0.181	0.795 ± 0.194	0.870 ± 0.145	0.860 ± 0.175	0.650 ± 0.247
	95% CI (Accuracy)	[0.737, 0.839]	[0.739, 0.849]	[0.831, 0.913]	[0.807, 0.907]	[0.577, 0.717]
	*p*-value (Acc/Bal)	0.015/0.333	0.030/0.030	0.005/0.005	0.005/0.005	0.095/0.095
	Sens/Spec	0.800/0.773	0.800/0.791	0.750/0.991	0.900/0.818	0.690/0.618

The table summarizes the predictive performance of the four supervised classifiers across all physiological trajectories. For each model–variable combination, five specific metrics are reported: (i) Accuracy (mean ± SD); (ii) Balanced Acc (mean ± SD) obtained from Repeated Stratified 5-Fold Cross-Validation with 10 repetitions (50 folds total); (iii) 95% CI (Accuracy); (iv) *p*-value (Acc/Bal), representing permutation-based *p*-values (computed with N = 1000 for Maximum Depth, K-Nearest Neighbors, and Nearest Centroid, and N = 200 for functional QDA) for both accuracy and balanced accuracy under the same cross-validation scheme; and (v) Sens/Spec, denoting the sensitivity and specificity derived from the accumulated confusion matrices across all folds. This structure highlights the substantial variability in classifier performance across variables, with Lactate and DBP consistently demonstrating stronger predictive value than Heart Rate or Glucose.

## Data Availability

The original contributions presented in this study are included in the article. Further inquiries can be directed to the corresponding author.
